# Study of Low-Temperature (Al)GaN on N-Polar GaN Films Grown by MOCVD on Vicinal SiC Substrates

**DOI:** 10.3390/ma18030638

**Published:** 2025-01-31

**Authors:** Yong Yang, Xianfeng Ni, Qian Fan, Xing Gu

**Affiliations:** Institute of Next Generation Semiconductor Materials, Southeast University, Suzhou 215123, China; 220222372@seu.edu.cn (Y.Y.); 103200036@seu.edu.cn (X.N.); 103200035@seu.edu.cn (Q.F.)

**Keywords:** N-polar GaN, MOCVD, surface morphology, low-temperature (Al)GaN, HEMTs

## Abstract

N-polar GaN HEMTs feature a natural back-barrier and enable the formation of low-resistance Ohmic contacts, with the potential to suppress short-channel effects and current collapse effects at sub-100 nm gate lengths, rendering them particularly promising for high-frequency communication applications. In this study, N-polar GaN films were grown on C-face SiC substrates with a 4° misorientation angle via MOCVD. By employing a two-step growth process involving LT-GaN or LT-AlGaN, the surface roughness of N-polar GaN films was reduced to varying degrees, accompanied by an improvement in crystalline quality. The growth processes, including surface morphology at each growth stage, such as the AlN nucleation layer, LT-GaN, LT-AlGaN, and the initial 90 nm HT-GaN, were investigated. The results revealed that a high V/III ratio and low-temperature growth conditions for the low-temperature layers, along with the introduction of a minor amount of Al, influenced adatom migration behavior and facilitated the suppression of step bunching. Suppressing step bunching during the initial growth stages was demonstrated to be critical for improving the surface quality and crystalline quality of N-polar GaN films. An N-polar GaN HEMT epitaxial structure was successfully achieved using the optimized surface morphology with a dedicated Fe-doped buffer process.

## 1. Introduction

To achieve the advantages of ultra-high data transmission rates, low latency, large bandwidth, and massive connectivity, the fifth-generation (5G) mobile communication technology requires the development of higher-frequency bands, particularly millimeter waves, which can support peak transmission rates exceeding 10 Gbps [1,2]. The design of large-scale multiple-input multiple-output (MIMO) antenna arrays in 5G systems demands the integration of more power amplifiers (PAs) into the radio frequency (RF) front-end, which makes PAs increasingly critical in 5G and beyond 5G systems compared to previous generations of communication technologies [3]. By leveraging the exceptional material properties of gallium nitride (GaN), including a wide bandgap (3.40 eV), high critical breakdown field strength (3 MV/cm), and the two-dimensional electron gas (2DEG) with high mobility (>2000 cm^2^/V·s) and carrier concentration (~1 × 10^13^ cm^−2^) provided by AlGaN/GaN heterojunctions, GaN high electron mobility transistors (HEMTs) are able to maintain high output power density at elevated frequencies. In addition, combined with the high thermal conductivity of the silicon carbide (SiC) substrates (3.7 W/cm·K), GaN HEMTs stand out from the competition with silicon (Si)-based laterally diffused metal oxide semiconductors (LDMOS) and gallium arsenide (GaAs)-based HEMTs, positioning them as the leading solution for 5G PA technology and beyond [4,5,6]. However, as operating frequency increases to the Ka- to W- band range, the gate length of GaN HEMTs must be reduced to the sub-micron scale, triggering or enhancing adverse phenomena such as short-channel effects and current collapse effects, thereby negatively impacting its RF performance [3,7,8]. Along the c-axis direction of the wurtzite structure, GaN material exhibits two different polarities: Ga-polarity and N-polarity. Due to the polarization direction difference, N-polar and Ga-polar HEMTs have opposite heterojunction sequences. In N-polar GaN HEMTs, the GaN channel resides above the AlGaN barrier. This heterojunction modification enables a reduction in the gate-to-channel distance without affecting the 2DEG concentration, thereby enhancing gate control to suppress short-channel effects. Additionally, a deep-recessed gate design can be employed to mitigate current collapse effects while maintaining an aspect ratio desirable for higher-frequency applications. Moreover, the N-polar HEMT structure incorporates a natural back-barrier and enables low Ohmic contact resistance, further improving device performance [8,9,10,11,12,13].

However, metal-organic chemical vapor deposition (MOCVD), widely used for large-scale commercial production, encounters significant challenges in N-polar GaN epitaxy due to poor film surface quality, typically characterized by numerous hexagonal hillocks. This issue arises primarily from the low migration ability of metal adatoms on the N-polar surface [14,15]. To address this issue, researchers have focused on using substrates with specific misorientation angles, such as sapphire or SiC substrates with a misorientation angle of 4° toward the m-plane or a-plane. The introduction of high-density narrow steps enhances the incorporation of metal adatoms into step edges or kinks, effectively suppressing the formation of hexagonal hillocks [16,17]. Nevertheless, the application of vicinal substrates significantly narrows the process window for achieving a step-flow growth mode in N-polar GaN films, often resulting in severe step bunching and surface coarsening. A coarse film surface hinders the formation of a clear and smooth AlGaN/GaN heterojunction interface in HEMT devices, reducing 2DEG mobility due to interface roughness scattering. Moreover, surface fluctuations along specific crystal directions induce significant anisotropy in the electrical performance of devices [18,19,20]. Currently, optimizing the surface morphology of N-polar GaN films on vicinal substrates emphasizes selecting an appropriate V/III ratio and employing two-step or multi-step growth processes. In general, a low V/III ratio corresponds to an increased migration rate of metal-adatom species. Li et al. [21] demonstrated that a high V/III ratio ensures higher nucleation density during the initial growth stage, leading to smoother surfaces. Brown et al. [17] were the first to achieve smooth N-polar GaN with high crystal quality on a carbon-face (C-face) SiC substrate misoriented by 4° toward the <10$\bar{1}$0> direction by the GaN two-step growth process. Won et al. [22] further pointed out that the reduction in compressive stress during the low-temperature growth process was conducive to the inhibition of step bunching. Recently, Zhang et al. [23] also proposed an optimization strategy involving multi-step temperature growth processes to overcome step bunching. Nevertheless, further investigations into the growth processes of N-polar GaN films, as well as the mechanism underlying the influence of key process parameters on the epitaxy growth quality, are still necessary.

In this work, based on the preliminarily optimized growth parameters, we grew N-polar GaN films on vicinal C-face SiC substrates with a misorientation angle of 4° toward the <11$\bar{2}$0> direction by MOCVD and introduced a low-temperature (LT) GaN or AlGaN through a two-step growth process. A comparative analysis was performed to examine the effects of the one-step and two-step growth process on the surface morphology and crystalline quality of N-polar GaN. The surface morphology at each growth stage, including the AlN nucleation layer, LT-GaN, LT-AlGaN, and the initial 90 nm high-temperature (HT) GaN layer, was systematically investigated. Based on the migration ability of metal adatoms, a detailed analysis was conducted on how the LT layer growth conditions could suppress step bunching, and the introduction of a minor amount of Al component further contributed to this suppression. Furthermore, based on the optimized surface morphology, we successfully obtained an N-polar GaN HEMT epitaxial structure with an Fe-doped buffer and controlled Fe-doping tail.

## 2. Materials and Methods

**Figure 1:**
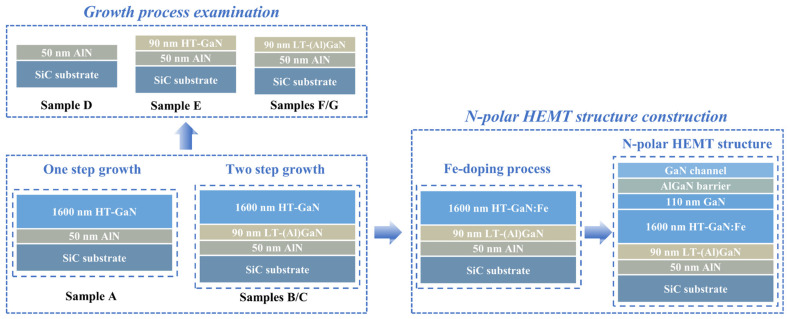
The investigation process flowchart of this experiment.

N-polar GaN films were grown on 4-inch C-face SiC substrates with a misorientation angle of 4° toward the <11$\bar{2}$0> direction by an AIXTRON G4 planetary MOCVD system. The rotation of the satellite wafer disks and the revolution of the graphite base ensured uniformity both within and between wafers, while the misorientation angle error of the substrates was controlled within ±0.1°. Before growth, a baking process was employed to clean the reaction chamber, minimizing contamination and ensuring experimental repeatability between runs. Trimethylaluminum (TMAl), trimethylgallium (TMGa), Ferrocene (CP_2_Fe), and ammonia (NH_3_) served as the precursors for Al, Ga, Fe, and N, respectively. The entire growth process was conducted in a pure H_2_ carrier gas environment, with the chamber pressure maintained at 50 mbar. Sample A adopted a one-step growth process. First, the SiC substrate was treated at 1200 °C in an H_2_ ambient for 720 s to remove surface contaminants and restore the atomic step morphology. Then, NH_3_ (0.67 mol/min) and TMAl (54.8 μmol/min) gas flow, with a high V/III ratio of 12,200, was introduced directly to grow a 50 nm AlN nucleation layer on the SiC substrate. The high temperature, high V/III ratio, and low-pressure AlN nucleation conditions reduced the risk of hexagonal inversion domain formation. Subsequently, the growth temperature was lowered to 1160 °C, and NH_3_ (0.36 mol/min) and TMGa (1.67 mmol/min) were introduced to grow approximately 1600 nm of HT-GaN under a V/III ratio of 215. Samples B and C adopted a two-step growth process, where 90 nm LT-GaN or LT-AlGaN was inserted between the AlN nucleation layer and HT-GaN at a growth temperature of 1000 °C, respectively, while the growth conditions for the other layers remained unchanged. The NH_3_ and TMGa flow rates for LT-GaN and LT-AlGaN were 0.91 mol/min and 553 μmol/min, respectively, with an additional flow of 21.9 μmol/min of TMAl introduced during the growth of LT-AlGaN. Due to the significantly lower flow rate of TMAl compared to TMGa, as well as the lower dissociative adsorption efficiency of TMAl [24,25], the growth rates of LT-GaN and LT-AlGaN were nearly identical. To further study the growth processes of the one-step and two-step growth process, four additional samples (Samples D–G) were prepared. Sample D consisted of only a 50 nm AlN nucleation layer, while Samples E, F, and G grew 90 nm HT-GaN, LT-GaN, and LT-AlGaN layers, respectively, based on Sample D. Since the performance parameters (such as crystal quality and morphology evolution) of N-polar GaN films are influenced by thickness, the thickness of each layer was strictly controlled. Furthermore, based on the optimized GaN buffer layer structure, an Fe doping process was applied to introduce deep-level acceptors that capture background electrons (with a CP_2_Fe flow rate of 0.012 μmol/min), thereby achieving high-resistivity GaN buffers. An additional unintentionally doped GaN layer was employed to eliminate the memory effect of Fe doping, ultimately facilitating the growth of an AlGaN/GaN heterojunction and enabling the realization of an N-polar GaN HEMT epitaxial structure. The process flowchart for this investigation is shown in Figure 1.

The N-polar GaN film growth process was monitored by an in situ reflectometer equipped with the MOCVD system, which employed a 635 nm wavelength laser to generate Fabry–Perot interference at both the epitaxial layer–substrate interface and the epitaxial layer surface to obtain information on the film thickness and growth mode. Macroscopic morphology was characterized using a 500× optical microscope (OM, SOPTOP MX8R, Ningbo, China), while microscopic morphology was examined with atomic force microscopy (AFM, SEMILAB, Budapest, Hungary) and scanning electron microscopy (SEM, Hitachi S-4700, Tokyo, Japan). The AFM operated in tapping mode with a scan range of 5 × 5 μm^2^. High-resolution X-ray diffraction (HRXRD, Malvern Panalytical X’Pert^3^ MRD, Almelo, The Netherlands) was used to perform symmetrical (002) and asymmetrical (102) X-ray omega (ω) rocking curve scanning to determine the full width at half maximum (FWHM) for crystal quality characterization, and omega-2theta (ω-2θ) scans were conducted to acquire structural and compositional information about the epitaxial films. A photoluminescence (PL, EtaMax PLATO-Series, Seoul, South Korea) system with a 266 nm wavelength laser was employed to verify the Al content in the LT-AlGaN. The Titanium/aluminum/nickel/Titanium (Ti/Al/Ni/Ti) stack was deposited using electron-beam evaporation (ULVAC ei-5z, Chigasaki, Japan) and without undergoing rapid thermal annealing (RTA) treatment. The electrical properties of N-polar GaN HEMT epitaxy wafers, including mobility, carrier concentration, and sheet resistance, were measured using a Lehighton Electronics contactless resistivity measurement system.

## 3. Results and Discussion

### 3.1. Al-Content Characterization of LT-AlGaN

**Figure 2:**
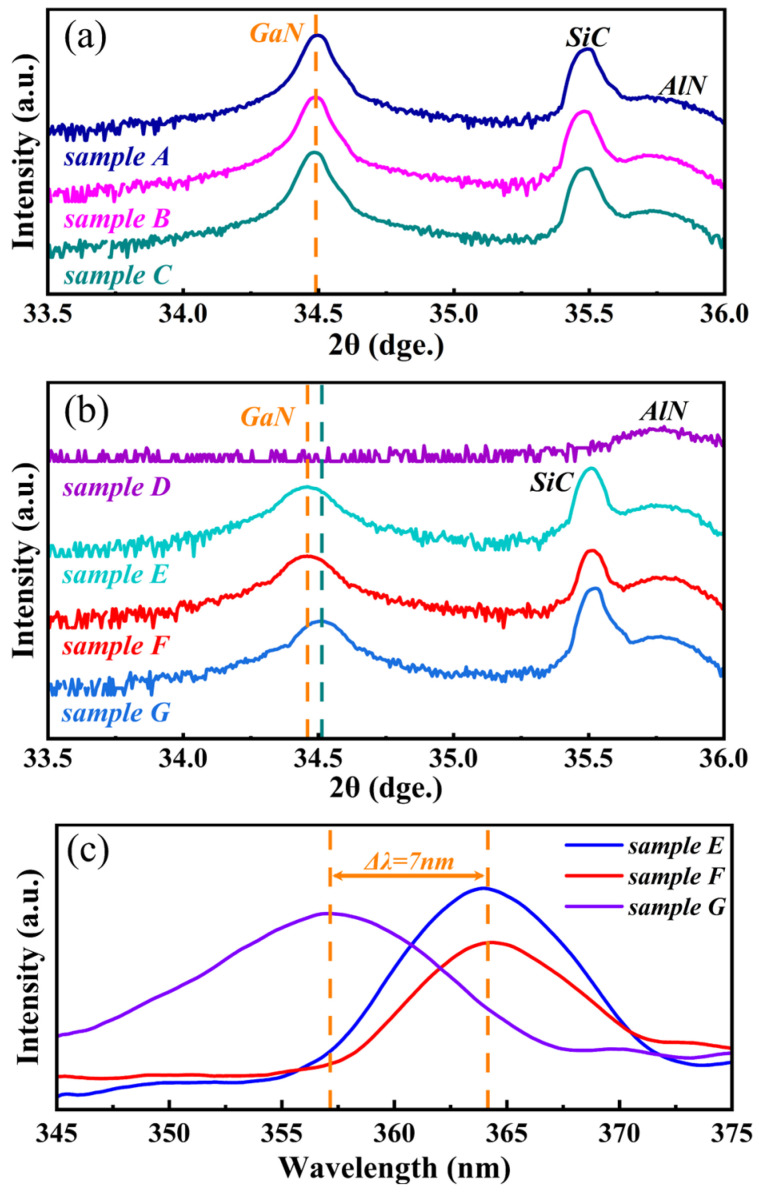
(**a**,**b**) HRXRD ω-2θ scan spectra of Samples A–C and Samples D–G in the 2θ range from 33.5° to 36.0°; (**c**) PL spectra for Samples E–G.

To verify the incorporation of Al atoms in the LT layer of Sample C and quantify their content, HRXRD ω-2θ scans in the 2θ range of 33.5° to 36.0° were performed for Samples A, B, and C, as shown in Figure 2a. Due to the use of vicinal substrates, the (002) diffraction peaks of GaN and AlN exhibited measurable offsets, deviating from the standard 2θ angles of 34.6° and 36.0°, respectively. However, the expected AlGaN diffraction peak was not observed to the right of the GaN diffraction peak in the scan spectrum of Sample C. This absence could result from a very low Al incorporation rate coupled with inferior crystalline quality, as indicated by the similar growth rates of LT layers in Samples B and C and the placement of LT layers beneath HT-GaN in the epitaxial structure, which could obscure their diffraction signals.

Further examination of the ω-2θ scan spectra for Samples E, F, and G (as shown in Figure 2b) revealed a slight shift in the diffraction peak of the LT layer in Sample G compared to those in Samples E and F. However, this shift may be attributed to slight variations in the misorientation angle of the vicinal SiC substrates, as the diffraction peak intensity of the SiC substrate is stronger than that of the LT layers, along with the test system’s calibration on the strongest diffraction peak. Accordingly, PL measurements were performed on Samples E, F, and G, as shown in Figure 2c. Samples E and F displayed prominent peaks around 364.5 nm, corresponding to a bandgap of 3.40 eV for GaN, while Sample G exhibited its strongest peak around 357.5 nm, indicating a 7 nm blue shift relative to Samples E and F, corresponding to an estimated bandgap of 3.47 eV. The Al content can be estimated using the following formula:

(1) $$E_{g}(x)=x\;\cdot {\;E}_{g,\mathrm{AlN}}+(1\;-\;x)\;\cdot {\;E}_{g,\mathrm{GaN}}-\;b\;\cdot \;x\cdot \;(1\;-\;x)$$

Here, E_g_(x) denotes the bandgap of AlGaN, where x denotes the Al content, and E_g,AlN_, E_g,GaN_ represent the bandgaps of AlN and GaN, taken as 6.20 eV and 3.40 eV, respectively. The bandgap bowing parameter, b, is taken as 0.353 [26]. The calculated Al content in the LT layer of Sample C is approximately 2.85%. Therefore, under the established growth parameters, a minor amount of Al was successfully incorporated into the LT layer of Sample C.

### 3.2. Polarity Examination

**Figure 3:**
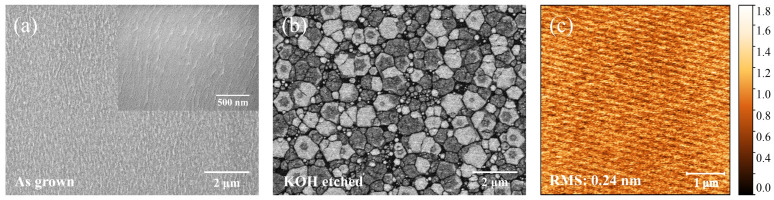
(**a**) SEM image of Sample C at a magnification of 10,000×, with an inset showing a magnification of 50,000×; (**b**) surface morphology of Sample C after 5 min of etching in 70 °C KOH solution; (**c**) AFM image of the SiC substrate surface after complete etching of the N-polar GaN films.

Studies have shown that inserting an LT-AlN layer after an N-polar HT-AlN nucleation layer can cause the polarity of the subsequent GaN growth to switch from N-polar to Ga-polar, whereas an LT-GaN insertion layer does not induce a comparable polarity transition [27]. Therefore, this study primarily examines the potential influence of the LT-AlGaN layer on the polarity of GaN grown on C-face SiC substrates. Hot potassium hydroxide (KOH) solution etching is a simple and efficient method for determining polarity. N-polar GaN, when etched with hot KOH solution, forms a hexagonal hillock structure with the {10$\bar{1}$$\bar{1}$} planes as the boundary, while Ga-polar GaN remains largely unaffected under the same conditions [28]. Figure 3a presents the surface of Sample C before etching, with the inset displaying an SEM image at 50,000× magnification, indicating a step-flow growth mode for the sample. After etching with hot KOH solution at 70 °C for 5 min, the surface exhibits hexagonal hillocks of various sizes Figure 3b). After thorough etching and physical cleaning, the SiC substrate surface shows no remaining GaN or AlN residues (Figure 3c), thereby confirming the N-polarity of the sample.

Furthermore, in a separate study of ours [29], the grown samples underwent chemical-mechanical polishing (CMP) with a weakly alkaline polishing slurry, resulting in a material removal rate (MRR) of approximately 600 nm/min. In contrast, the MRR of Ga-polar GaN is typically only a few tens of nanometers per hour [30,31], indirectly corroborating that the samples grown in this article are N-polar GaN films, which exhibit higher chemical activity.

### 3.3. Surface Morphology

**Figure 4:**
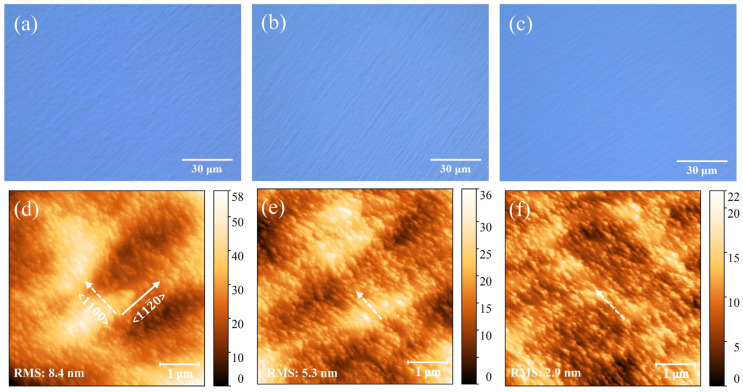
(**a**–**c**) OM images of Samples A–C at a magnification of 500×; (**d**–**f**) AFM images of Samples A–C in a 5 × 5 μm^2^ area, where solid and dashed white arrows represent the <11$\bar{2}$0> and <1$\bar{1}$00> crystallographic directions, respectively.

As shown in Figure 4a–c, due to the combined effects of the narrow steps of the vicinal SiC substrates and the optimized MOCVD growth conditions, no hexagonal hillocks were observed in Samples A, B, and C at 500× magnification in OM imaging. However, all the samples exhibited striped undulations in the microscope images. In contrast to the rotational displacement of 30° between GaN and sapphire in-plane lattices, the SiC substrates and GaN maintain a strictly aligned epitaxial orientation relationship [13,23]. These macroscopic undulations are distributed along the miscut direction of <11$\bar{2}$0> and are perpendicular to the <1$\bar{1}$00> direction. In contrast to Samples B and C, Sample A exhibits more disordered macroscopic stripe features, accompanied by frequent and distinct triangular undulations, which are also evident in the AFM images shown in Figure 4d. The surface of Sample A displays significant protrusions and depressions, with a peak-to-valley height difference of 58 nm and a root mean square (RMS) roughness of 8.4 nm within the 5 × 5 μm^2^ test area. The AFM images of Samples B and C (as shown in Figure 4e,f) exhibit more uniform and smoother surfaces, with RMS values of 5.3 nm and 2.9 nm, respectively. The results indicate that the use of LT insertion layers significantly improved surface flatness, with the incorporation of a minor amount of Al atoms in the LT-AlGaN layer contributing further to the reduction in surface roughness.

**Figure 5:**
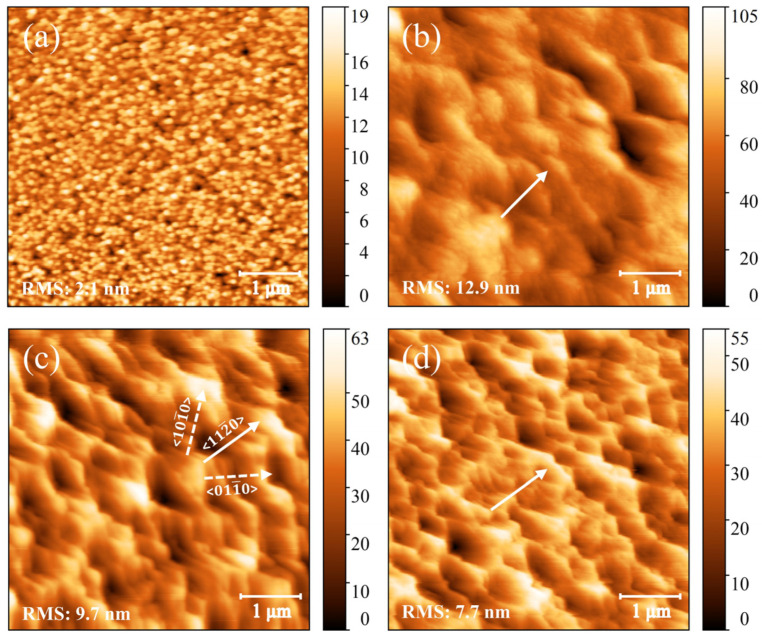
(**a**–**d**) AFM images of Samples D–G in a 5 × 5 μm^2^ area, where the solid white arrows represent the <11$\bar{2}$0> crystallographic direction, and the dashed white arrows represent the <10$\bar{1}$0> or <01$\bar{1}$0> crystallographic directions.

To investigate the effects of LT-GaN and LT-AlGaN on surface morphology and explore the growth process of N-polar GaN films, the surface morphologies of the AlN nucleation layer, initial 90 nm HT-GaN, LT-GaN, and LT-AlGaN layers (i.e., Samples D–G) were analyzed, as shown in Figure 5. The morphology of the AlN nucleation layer, as shown in Figure 5a, predominantly exhibits an island-like growth mode. The average size of the nucleation islands is approximately 100 nm, uniformly and diffusely distributed on the SiC substrate. In contrast, during Ga-polar GaN epitaxy, the AlN nucleation layer tends to adopt a quasi-two-dimensional growth mode [32]. The formation of the island-like growth mode in the N-polar AlN nucleation layer is likely caused by the higher activation energy for Al adatom migration on the N-polar surface, combined with the high V/III ratio required for N-polar AlN nucleation, which decreases the mobility of Al adatoms.

**Figure 6:**
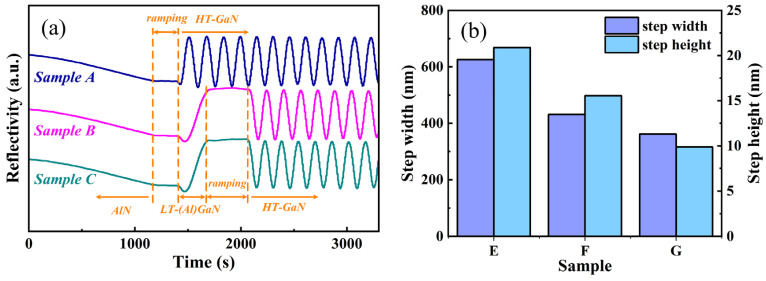
(**a**) In situ reflectivity curves during MOCVD growth for Samples A–C, where the entire growth process for Sample A is shown, and the HT-GaN growth process for Samples B and C is shown in part; (**b**) histogram of the average step width and height statistics from the AFM images of Samples E-G shown in Figure 5b–d.

During the subsequent 90 nm growth of N-polar GaN or AlGaN, the growth mode rapidly transitioned from island growth to layer-by-layer growth, as shown in Figure 5b–d. This transition is further corroborated by the in situ reflectance curves, as shown in Figure 6a. During AlN nucleation layer growth, the reflectance continuously decreased, possibly due to interference from reflectance itself and the relatively lower growth rate, as well as the island growth mode of the AlN nucleation layer. The reflectance then briefly dipped in the early stages and rapidly increased to saturation during HT-GaN or LT-(Al)GaN growth. This behavior contrasts with the typical prolonged island-like growth mode observed in LT Ga-polar GaN. These findings are consistent with comparative experiments by Takashi et al. [33], which demonstrated that N-polar GaN undergoes lateral growth before Ga-polar GaN under LT conditions.

The surface morphology of Samples E–G exhibits typical triangular step features, which are contingent upon the misorientation direction of the SiC substrates. Due to the hexagonal wurtzite crystal structure of GaN, there are two types of atomic step edges: type-A and type-B. Each atom at a type-A step edge has two dangling bonds, whereas each atom at a type-B step edge has only one dangling bond. Consequently, the growth rate of the type-A step edge is significantly faster than that of the type-B step edge. Notably, these two types of atomic step edges alternate in stacking along the <0001> or <000$\bar{1}$> direction. During GaN crystal growth, the overall migration rate of the step edge is limited by the slower-growing type-B step edge, ideally leading to the formation of steps with a double bilayer height of 5.2 Å [34,35]. When the N-polar GaN grows on the C-face SiC substrates with a misorientation toward the <11$\bar{2}$0> direction, the step edges perpendicular to the <10$\bar{1}$0> and <01$\bar{1}$0> directions differ within the same atomic plane, forming a triangular step morphology with an angle of 120°. In contrast, when the GaN epitaxy grows on vicinal C-face SiC substrates toward the <10$\bar{1}$0> direction, only one type of atomic step edge exists within the same atomic plane. This isotropic characteristic leads to more stable GaN growth. Consequently, compared to the growth on vicinal SiC substrates oriented toward the a-plane or vicinal sapphire substrates oriented toward the m-plane, N-polar GaN grown on vicinal SiC substrates oriented toward the m-plane or vicinal sapphire substrates oriented toward the a-plane typically exhibits a more ordered and smoother surface morphology [36].

Moreover, Samples E-G exhibit varying degrees of step bunching, as observed in the AFM morphology, with Sample E showing the most severe and Sample G the mildest effects. The average step width and height statistics for the three samples are shown in Figure 6b. The average step widths for Samples E, F, and G are 625.6, 431.1, and 361.6 nm, respectively, while the average step heights are 20.9, 15.6, and 9.9 nm. Epitaxial growth modes are usually closely tied to the migration behavior of adsorbed atoms. Typically, when the migration distance of adatoms exceeds the step width, a step-flow growth mode forms, resulting in a smoother surface. However, when III-nitrides epitaxy is performed on vicinal substrates with a larger misorientation angle, a different growth mode known as step bunching is frequently observed. This mode arises because adatoms preferentially incorporate into the lower atomic steps rather than the upper ones, causing the wider upper atomic steps to expand further while the narrower lower atomic steps gradually bunch together, thus leading to surface roughening [37]. For a fixed misorientation angle (i.e., a determined step width), the transition between step-flow growth and step bunching growth can be controlled by modifying the migration ability of adatoms. According to the model proposed by Isaac Bryan et al. [38], increasing the vapor supersaturation (i.e., reducing the migration ability of adsorbed atoms) is beneficial for inhibiting step bunching. In this study, pure H_2_ was used as the carrier gas, with *F* = H_2_/(H_2_ + N_2_) = 1. Under this circumstance, the vapor supersaturation increases monotonically with an increase in the V/III ratio [39], and it also increases as the growth temperature decreases [18]. Since the growth temperature of LT-GaN is lower than that of HT-GaN (1000 °C vs. 1160 °C), and its V/III ratio is higher than that of HT-GaN (1707 vs. 215), the Ga supersaturation of LT-GaN is significantly higher than that of HT-GaN. This results in a reduced step bunching effect in Sample F compared to Sample E. Further comparison between Samples F and G shows that, due to the higher bond energy of Al-N compared to Ga-N [40], Al adatoms have lower mobility on the growth surface than Ga adatoms. The introduction of a minor amount of Al component reduces the average migration ability of metal adatoms without significantly affecting the Ga supersaturation, thereby further alleviating step bunching.

As the epitaxial thickness increases, step bunching tends to saturate or diminish, and adatoms may nucleate and grow on large step-free terraces between the bunches [37]. A comparison of Figure 5b–d and Figure 4d–f reveals that the macroscopic triangular steps on the initial (Al)GaN underlying layers vanish, giving way to microscopic step structures on a broader undulating surface, as also shown in the inset of Figure 3a. This observation suggests that the subsequent N-polar GaN likely undergoes a step-flow growth mode. Throughout the entire N-polar GaN epitaxial growth process, under the condition of vicinal substrate with a misorientation angle of 4°, the initial GaN layer experiences significant step bunching. The introduction of LT-GaN or LT-AlGaN layers effectively suppresses this step bunching effect, establishing a better-quality surface foundation for subsequent N-polar GaN growth. The RMS values decrease progressively from Sample C to Sample A. Therefore, suppressing step bunching during the early growth stages is crucial for achieving smooth N-polar GaN films.

### 3.4. Crystalline Quality

**Figure 7:**
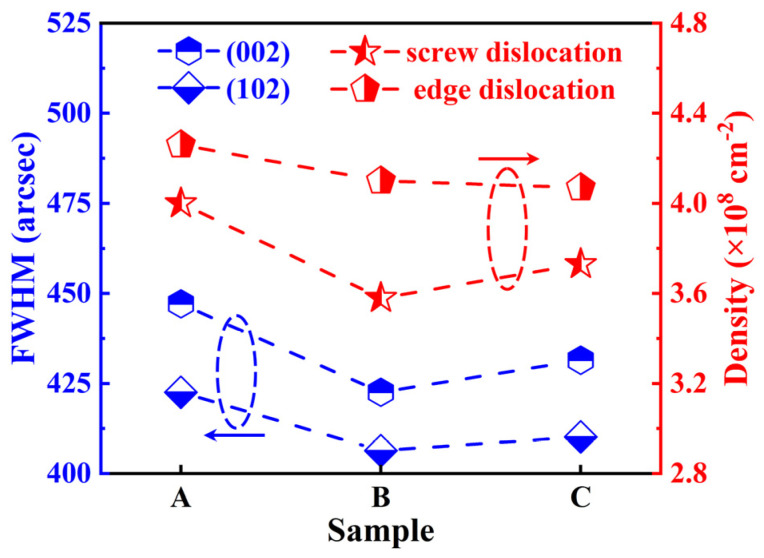
HRXRD rocking curve FWHMs (blue symbols) of symmetrical (002) and asymmetrical (102) scans for Samples A–C, along with the corresponding dislocation density (red symbols).

Symmetric (002) and asymmetric (102) X-ray omega rocking curve scans were performed on Samples A–C. The broadening of the (002) diffraction peak arises typically from screw dislocations, whereas the broadening of the (102) diffraction peak results typically from mixed or edge dislocations. The calculation formula linking the FWHM of diffraction peaks to dislocation density is described in the literature [41]. Figure 7 presents the X-ray omega rocking curve FWHM values and the corresponding dislocation densities for Samples A–C on the (002) and (102) planes. The total dislocation densities of the three samples are comparable, measured at 8.28 × 10^8^, 7.68 × 10^8^, and 7.80 × 10^8^ cm^−2^, respectively.

In a conventional two-step growth process, LT-GaN is typically grown at temperatures ranging from 500 °C to 600 °C [33,42,43,44]. The crystalline quality of the subsequently grown HT-GaN is significantly influenced by the growth parameters of LT-GaN and the annealing conditions, which determine the quality and morphology of the GaN nucleation islands [45]. In N-polar GaN epitaxy on vicinal SiC substrates, the growth temperature of the LT layers is typically around 1000 °C, deviating from conventional LT-GaN growth conditions [17,22,41]. Under such conditions, the island-like growth mode becomes unsustainable and transitions rapidly into a layer-by-layer growth mode. Consequently, the overall crystalline quality of the epitaxial film is predominantly governed by the immediate growth conditions. Owing to the consistent growth conditions of HT-GaN, the crystalline quality of the three samples remains comparable. Nevertheless, the introduction of LT-GaN and LT-AlGaN layers slightly reduces the total dislocation density, due to the high V/III ratio conditions employed during their growth. The LT layers, compared with HT-GaN, have a higher V/III ratio, providing a greater abundance of Ga vacancies. Studies [46,47,48] suggest that dislocations bend and annihilate upon interacting with these Ga vacancies. Therefore, the introduction of the LT layer growth process in Samples B and C effectively reduced the total dislocation density and improved the overall crystalline quality of the epitaxial films.

### 3.5. Construction of N-Polar GaN HEMT Epitaxy Structure

**Figure 8:**
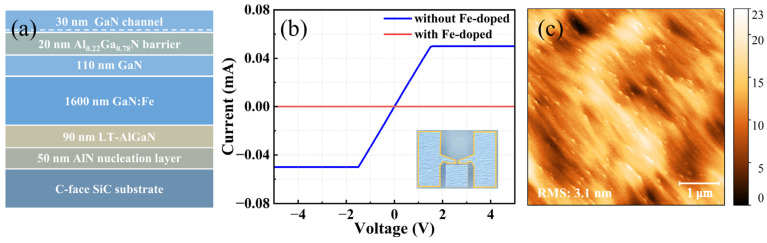
(**a**) N-polar GaN HEMT epitaxial structure; (**b**) I-V test curves for N-polar GaN buffer with (red line) and without (blue line) Fe doping; the test structure is the metal electrode surrounded by the yellow box in the bottom right illustration, with a minimum distance of 13 μm between the two electrodes; (**c**) AFM images of the top GaN channel surface of the N-polar GaN HEMT epitaxial wafer.

Building upon the aforementioned work, we commenced the development of an N-polar HEMT epitaxy structure, with the full epitaxy structure depicted in Figure 8a. The N-polar GaN buffer adopts the structure of Sample C, which offers smooth surface morphology and high crystalline quality. On the N-polar surface, oxygen atoms impinging on group-V sites can bond with triple Ga atoms, whereas on the Ga-polar surface, only one single bond with a Ga atom is formed. Consequently, oxygen atoms are less likely to desorb from the N-polar growth surface, allowing the oxygen concentration to easily reach the order of 10^18^ cm^−3^ [49,50]. The elevated oxygen concentration introduces numerous background carriers, compromising the semi-insulating characteristics of the buffer layer. A Ti/Al/Ni/Ti stack with thicknesses of 5/70/50/10 nm was deposited on Sample C through electron-beam evaporation to serve as the contact electrode. The electrode layout is depicted within the yellow box in the inset of Figure 8b, with a minimum spacing of 13 μm. Under the applied test voltage of −5 to +5 V, the I-V curve is shown as the blue curve in Figure 8b, with a resistance of only 29.8 Ω, confirming the characteristics of an N-type semiconductor. The saturation current at high voltage corresponds to the protection current set by the test system. Fe doping was implemented to introduce deep-level acceptors, ensuring the semi-insulating properties of the buffer. As a result of Fe doping, the resistance increased to approximately 10^6^ Ω under the same testing conditions. However, due to the strong memory effect of Fe doping, the doping does not immediately cease after turning off the Fe precursor. To prevent Fe atoms from diffusing into the channel region, an additional 110 nm unintentionally doped GaN layer with a low oxygen impurity concentration was grown under a high V/III ratio condition to eliminate Fe doping tails. Secondary ion mass spectroscopy (SIMS) analysis revealed that the oxygen impurity concentration in this layer was 5 × 10^17^ cm^−3^. Subsequently, a 20 nm thick Al_0.22_Ga_0.78_N barrier and a 30 nm thick GaN channel were grown to introduce strong polarization and facilitate the generation of a 2DEG. The AFM morphology of the GaN channel surface is shown in Figure 8c, which exhibits a similar surface roughness to that of the buffer. The mobility, carrier concentration, and sheet resistance of the N-polar GaN HEMT epitaxial wafer were measured to be 727.55 cm^2^/V·s, 1.06 × 10^13^ cm^−2^, and 809.52 Ω/sq, respectively, using a contactless resistivity measurement system.

## 4. Conclusions

In summary, we report the epitaxial growth of N-polar GaN films on C-face SiC substrates with a misorientation angle of 4° toward the <11$\bar{2}0$> direction by MOCVD, and investigate the effects of the one-step and two-step growth process on the surface morphology and crystalline quality of N-polar GaN films. The incorporation of LT-GaN and LT-AlGaN successfully reduced the RMS roughness of N-polar GaN within a 5 × 5 μm^2^ area from 8.4 nm to 5.3 nm and 2.9 nm, respectively, while the total dislocation density also exhibited a slight decrease. The surface morphology at each growth stage, including the AlN nucleation layer, LT-GaN, LT-AlGaN, and the initial 90 nm HT-GaN, was thoroughly examined. The AlN nucleation layer showed an island-like growth mode, characterized by uniformly distributed nucleation islands that provided sufficient cores for subsequent GaN growth. In both LT and HT layers, the growth mode rapidly transitioned from island-like growth mode to layer-by-layer growth mode, accompanied by varying degrees of step bunching. The high V/III ratio and low-temperature conditions of the LT layer significantly increased Ga vapor supersaturation and reduced the migration ability of Ga adatoms, thereby partially inhibiting step bunching. The addition of a minor amount of the Al component, possessing high migration activation energy, further diminished metal adatom migration, thereby alleviating step bunching. The mitigation of step bunching in the underlying layer establishes a higher-quality surface foundation for subsequent N-polar GaN growth, facilitating improved surface morphology. Additionally, based on the optimized surface morphology, Fe doping was conducted to achieve a high-resistivity buffer, while an unintentionally doped GaN layer with a low oxygen impurity concentration was grown to eliminate Fe doping tails, culminating in the successful realization of the N-polar GaN HEMT epitaxial structure.

## Data Availability

The original contributions presented in this study are included in the article. Further inquiries can be directed to the corresponding author.
